# *PROM1*/CD133 marks a proliferative stem cell-like population of blasts in *KMT2A* rearranged infant ALL^∗^

**DOI:** 10.1182/bloodadvances.2024015185

**Published:** 2025-07-05

**Authors:** Joe Wilson Cross, Lucy Field, Alastair Smith, Emily Neil, Lucy Hamer, Thomas Jackson, Natalina Elliott, Siobhan Rice, Nicholas Crump, Joe Harman, Rebecca E. Ling, Qingqing Wu, Nouhad El Ouazzani, Rebecca Thomas, Sarah Inglott, Jack Bartram, Owen Smith, Jonathan Bond, Irene Roberts, Thomas A. Milne, Anindita Roy

**Affiliations:** 1 Department of Paediatrics, University of Oxford, Oxford, United Kingdom; 2 MRC Molecular Haematology Unit, MRC Weatherall Institute of Molecular Medicine, University of Oxford, Oxford, United Kingdom; 3 Centre for Haematology, Department of Immunology and Inflammation, Hugh and Josseline Langmuir Centre for Myeloma Research, Imperial College London, London, United Kingdom; 4 Department of Haematology, Great Ormond Street Hospital, London, United Kingdom; 5 School of Medicine, Trinity College, University of Dublin, Dublin, Ireland; 6 School of Medicine, Systems Biology Ireland, School of Medicine, University College Dublin, Dublin, Ireland; 7 National Children's Cancer Service, Children's Health Ireland at Crumlin, Dublin, Ireland

**TO THE EDITOR:**

Infant acute lymphoblastic leukemia (iALL) is an aggressive disease that remains a major clinical challenge.^1^, ^2^, ^3^ In 70% to 80% of iALL, translocations of *KMT2A* gene, most commonly with *AFF1* [t(4;11) (q21;q23)], produce an oncogenic fusion protein which recruits a large protein complex, "rewriting" epigenetic marks to alter expression of target genes.^4^, ^5^, ^6^ Understanding the KMT2A fusion protein complex has been vital for identifying targets such as menin for novel therapies.^7^ Investigation of genes regulated by KMT2A::AFF1 also remains an important goal.

Previously, we showed that one of the most profoundly dysregulated genes in *KMT2A*::*AFF1* leukemia was *PROM1*, which encodes the cell surface glycoprotein CD133.^8^ Proliferation of *KMT2A*::*AFF1* ALL cell lines was highly dependent on CD133 expression and *PROM1*/CD133 was expressed at significantly higher levels in *KMT2Ar* ALL than in *KMT2A* germ line ALL.^8^, ^9^, ^10^

CD133 has been identified as a marker of stem cells in many cancers^11^^,^^12^ including leukemia,^13^^,^^14^ and is also expressed on normal hematopoietic stem and progenitor cells.^15^ Expression of CD133 in samples from patients with *KMT2A*::*AFF1* ALL, is often heterogenous and the mechanisms by which CD133 contributes to leukemia biology are unclear.^16^

Here we investigate the function of CD133 in *KMT2A*::*AFF1* iALL using a primary human fetal liver–derived model of *KMT2A*r leukemia^17^ that recapitulates the pattern of CD133 expression observed in patient samples. We found that CD133 marks an aggressive population of blasts with a stem cell-like signature in *KMT2Ar* ALL. This provides a rationale for targeting CD133 by pharmacological or immunotherapy-based approaches alongside other treatment modalities.

Single guide RNA (Synthego) triplets targeting the *PROM1* start codon were used for *PROM1* knockout (*PROM1* KO) (supplemental Table 1). Cells were electroporated with Cas9-only or Cas9-single guide RNA complexes using a Neon Transfection System (Thermo Fisher) at 1600 V, 10 milliseconds, 3 pulses and recovered overnight in SFEM II (Stemline) supplemented with 10% fetal bovine serum (Invitrogen), 10 nM interleukin-3 and 5 nM interleukin-7 (Peprotech).

For ^CRISPR^*KMT2A::AFF1* blast coculture, MS5 stromal layers were prepared as described previously.^17^ About 2000 ^CRISPR^*KMT2A*::*AFF1* blasts were seeded/well in StemSpan SFEM II (serum free medium for culture and expansion of hematopoietic cells) supplemented as above and incubated at 37°C per 5% CO_2_ with twice-weekly half-volume medium changes. Flow-cytometric readouts were performed weekly with replating onto fresh stromal layers (supplemental Table 2).

For ^CRISPR^*KMT2A::AFF1* blast xenograft assays, CD19^+^ blasts from the bone marrow of mice that developed ^CRISPR^*KMT2A*::*AFF1* ALL were used for xenotransplantation assays, either after sorting for CD133^+^ and CD133^–^ fractions, or after *PROM1* KO. About 30 000 to 60 000 blasts were injected via tail vein into sublethally irradiated NSG mice, and the mice were monitored as described previously.^17^

Additional methods are described in the supplemental Material.

We examined expression of *PROM1* and CD133 in the human fetal liver–derived ^CRISPR^*KMT2A*::*AFF1* iALL model.^17^ *PROM1* expression was significantly increased in CD19^+^ bone marrow blasts from mice with ^CRISPR^*KMT2A*::*AFF1* leukemia compared to Cas9-only controls (Figure 1A; *P* = .02), and correlated strongly with CD133 cell surface protein expression as measured by flow cytometry (Figure 1B). The pattern of CD133 expression in ^CRISPR^*KMT2A*::*AFF1* leukemias was variable, as in patient samples (Figure 1C).

**Figure 1.**
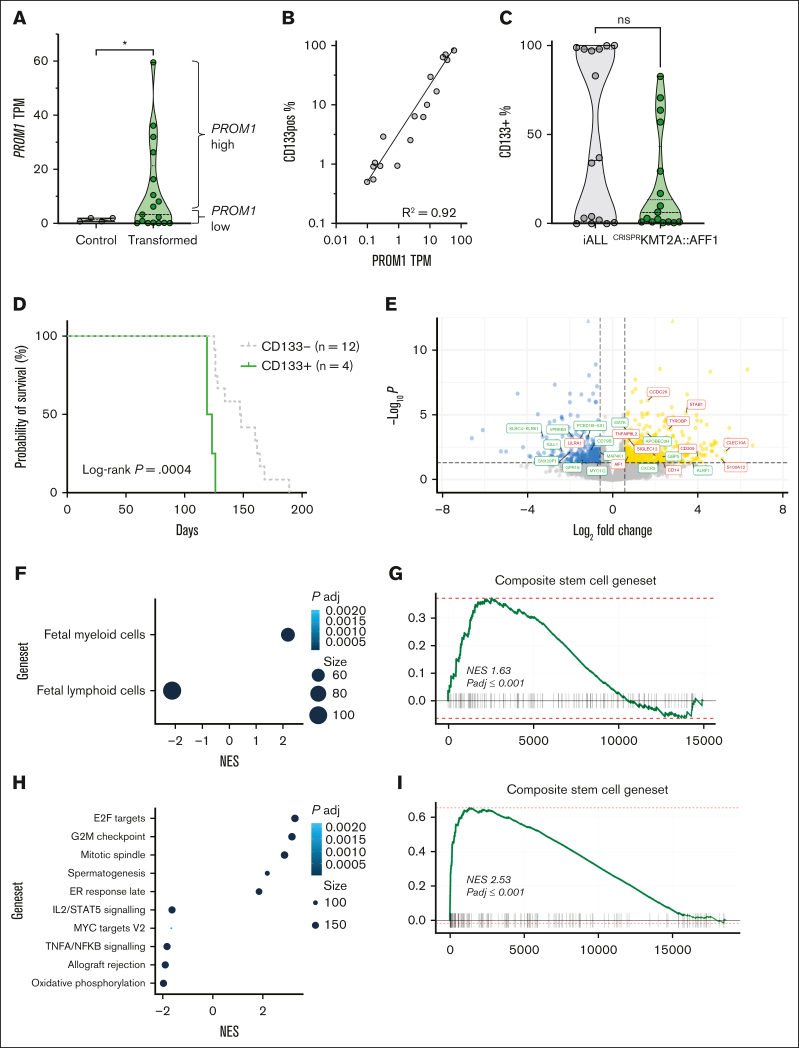

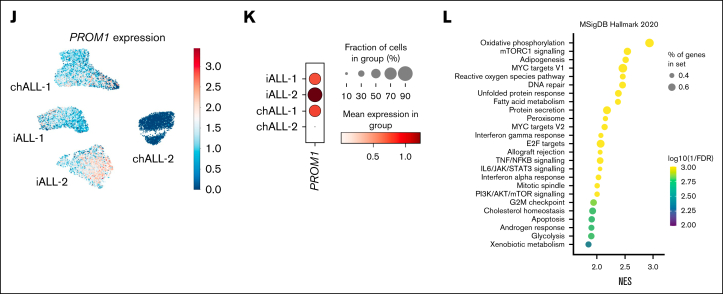
**The ^CRISPR^*KMT2A*::*AFF1* iALL model recapitulates variation in CD133 expression, and CD133 status correlates with differences in functional and molecular signatures.** (A) *PROM1* TPM from hCD45^+^CD19^+^ cells from bone marrow of NSG mice transplanted with Cas9-only (control, n = 4) and ^CRISPR^*KMT2A*::*AFF1* leukemias (transformed, n = 17) fetal liver CD34^+^ cells (*P* = .02). *PROM1* high (above median) and low (below median) leukemias are indicated by brackets. (B) Correlation of *PROM1* gene expression and CD133 protein expression by flow cytometry for ^CRISPR^*KMT2A*::*AFF1* leukemias (n = 17, *R*^2^ = 0.92). (C) CD133 positivity in CD19^+^ blasts in samples of patients with iALL at diagnosis (n = 11) and relapse (n = 5) compared to ^CRISPR^*KMT2A*::*AFF1* leukemias (n = 17). (D) Survival curves of NSG mice with CD133^+^ (defined as >20% of blasts CD133^+^) or CD133^–^ primary ^CRISPR^*KMT2A*::*AFF1* leukemias (n = 4 CD133^+^, n = 12 CD133^–^, log-rank *P* = .0004). (E) Volcano plot of differential gene expression between *PROM1* high and low ^CRISPR^*KMT2A*::*AFF1* leukemias. Genes with adjusted *P* < .05 and log-fold change >0.58 or less than –0.58 are shown in yellow and blue, respectively. Myeloid/lymphoid program associated genes are labelled in red and green, respectively. (F) Gene set enrichment analysis (GSEA) reaching statistical significance (false discovery rate [FDR] 0.05) from fetal entries in MSigDB C8 (cell type signature gene sets^18^), ^CRISPR^*KMT2A*::*AFF1 PROM1* high vs low. (G) GSEA of a core stem cell gene set,^19^^CRISPR^*KMT2A*::*AFF1 PROM1* high vs low (normalized enrichment score = 1.63, adjusted *P* ≤ .001). (H) GSEA reaching statistical significance (FDR, 0.05) from entries in MSigDB H1 (hallmark gene sets), ^CRISPR^*KMT2A*::*AFF1* CD133^+^ vs CD133^–^ blasts. (I) GSEA of a composite core stem cell gene set,^19^^CRISPR^*KMT2A*::*AFF1* CD133^+^ vs CD133^–^ blasts (normalized enrichment score = 2.88, adjusted *P* ≤ .001). (J) Uniform manifold approximation and projection (UMAP) of single-cell transcriptomic data of CD19^+^ blasts from 4 samples of patients with *KMT2A*::*AFF1*, displaying log1p (normalized *PROM1* gene expression) (n = 2, chALL; n = 2, iALL, total of 14 927 cells). (K) Dot plot showing *PROM1* expression pattern at a single-cell level in the 4 *KMT2A*r patient samples in panel J. (L) Top 25 most enriched gene set entries in MSigDB H1 (hallmark gene sets) by GSEA, *PROM1*^*+*^ vs *PROM1*^*–*^ blasts from *KMT2A*::*AFF1* primary patient samples analyzed by single-cell (sc) RNA-sequencing in panel J. AKT, protein kinase B; chALL, childhood ALL; IL, interleukin; mTORC1, mammalian target of rapamycin complex 1; mTOR, mammalian target of rapamycin; ns, not significant; NES, normalized enrichment score; padj, *P* adjusted; PI3K, phosphoinositide 3-kinase; TPM, transcripts per million; TNFA, tumor necrosis factor alpha.

To interrogate how *PROM1/*CD133 expression affects the biology of *KMT2A*::*AFF1* iALL, we compared CD133^+^ and CD133^–^^CRISPR^*KMT2A*::*AFF1* ALL. NSG mice developing primary CD133^+^^CRISPR^*KMT2A*::*AFF1* leukemia (n = 4) had significantly shorter survival than mice developing CD133^–^ (n = 12) leukemia (Figure 1D; 119 vs 140.7 days median, *P* = .0004). RNA sequencing of CD19^+^ blasts showed enrichment of fetal myeloid programs^18^ and a composite stem cell gene set^19^ along with reduced expression of lymphoid programs^18^ in *PROM1*-high ^CRISPR^*KMT2A*::*AFF1* ALL (Figure 1E-G; supplemental Figure 1A-B; supplemental Table 3). Some of these features were also seen in primary patient samples^10^ when comparing *PROM1*-high and *PROM1*-low *KMT2A*::*AFF1* ALL (supplemental Figure 1C).

As CD19^+^ blasts from the same leukemia (both in patients and ^CRISPR^*KMT2A*::*AFF1* ALL) have variable expression of CD133, we prospectively isolated CD133^+^ and CD133^–^ blasts from ^CRISPR^*KMT2A*::*AFF1* leukemias to determine the correlation between CD133 expression and the molecular and functional characteristics of *PROM1-*high leukemias (supplemental Figure 2A). RNA sequencing showed that of the 162 upregulated and 75 downregulated differentially expressed genes (supplemental Table 4) in CD133^+^ blasts, most enriched gene sets were associated with cell cycling, cell division, survival, and proliferation, as well as stemness (Figure 1H-I).

We generated single-cell transcriptomic data from CD19^+^ blasts fluorescence-activated cell sorted (FACS) from 4 samples of patient with *KMT2Ar* ALL (2 infants: iALL; 2 childhood) (supplemental Figure 2B). Childhood ALL-2 showed no *PROM1* expression, whereas the other 3 samples demonstrated a proportion of cells with heterogeneous levels of *PROM1* expression (Figure 1J-K), which correlated well with protein expression (supplemental Table 5). *PROM1*^*+*^ blasts enriched for cell proliferation signatures (myelocytomatosis family/early region 2 binding factor targets, mammalian target of rapamycin complex 1 signaling) in comparison to *PROM1*^*–*^ blasts (Figure 1L; supplemental Figure 2C), similar to CD133^+^^CRISPR^*KMT2A*::*AFF1* blasts. However, *PROM1*^*+*^ patient blasts also enriched for OXPHOS pathway, unlike CD133^+^^CRISPR^*KMT2A*::*AFF1* blasts.

In keeping with these findings, FACS CD133^+^^CRISPR^*KMT2A*::*AFF1* blasts underwent ∼100-fold higher expansion in vitro compared to CD133^*–*^ blasts from the same leukemia (Figure 2A). More CD133^+^ blasts were in S/G2/M phase after 7 days in vitro *(*Figure 2B-C; 10.0% vs 5.8%, *P* = .003), whereas fewer were in subG1 phase (Figure 2D; 1.7% vs 4.5%, *P* = .01) compared to CD133- blasts, consistent with increased cell cycling and apoptosis resistance. Confirming these findings in vivo, mice transplanted with CD133^+^^CRISPR^*KMT2A*::*AFF1* blasts showed more rapid engraftment (supplemental Figure 2D) and reduced survival (Figure 2E; 71 vs 105 days median, *P* = .0033).

**Figure 2.**
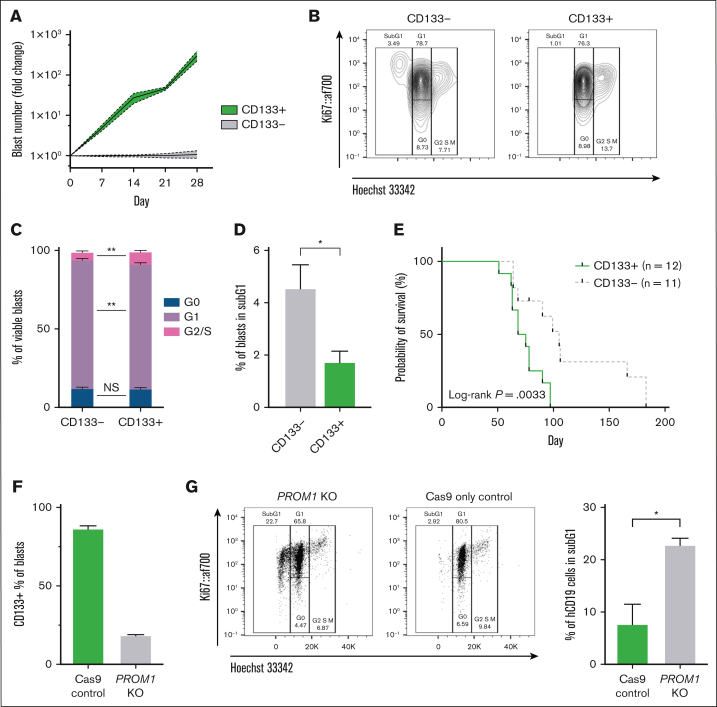

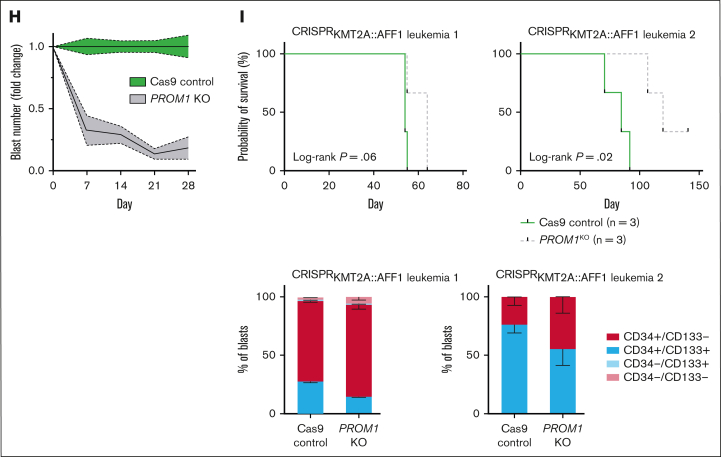
**CD133 expression marks KMT2A::AFF1 blasts with higher proliferative potential.** (A) Proliferation of CD133^+^ and CD133^–^^CRISPR^*KMT2A*::*AFF1* blasts in MS5 coculture. Data shown as fold change in absolute blast count of CD133^+^ cells compared to CD133^–^ cells at each time point (n = 4 biological replicates, mean values with error bars = standard error of the mean [SEM]). (B) Representative flow plots from cell cycle analysis of CD133^–^ (left) and CD133^+^ (right) ^CRISPR^*KMT2A*::*AFF1* blasts on day 7 of coculture. (C) Quantification of cell cycle status of CD133^–^ and CD133^+^^CRISPR^*KMT2A*::*AFF1* blasts on day 7 of coculture (n = 4 biological replicates, data shown as mean ± SEM). (D) Percentage of CD133^–^ and CD133^+^^CRISPR^*KMT2A*::*AFF1* blasts in sub-G1 phase on day 7 of coculture (n = 4 biological replicates, data shown as mean, error bars = SEM). (E) Survival curves of NSG immunodeficient mice transplanted with CD133^+^ or CD133^–^^CRISPR^*KMT2A*::*AFF1* leukemia cells (4 biological replicates, n = 12 CD133^+^ animals, n = 11 CD133^–^ animals, log-rank *P* = .0033). (F) Efficiency of *PROM1* KO in ^CRISPR^*KMT2A*::*AFF1* blasts assessed by CD133 surface protein expression by flow cytometry, 7 days postelectroporation (error bars = standard deviation). (G) Left: representative flow plots from cell cycle analysis of *PROM1* KO and control ^CRISPR^*KMT2A::AFF1* blasts 72 hours after electroporation. Right: percentage of *PROM1* KO and control ^CRISPR^*KMT2A*::*AFF1* blasts in sub-G1 phase 72 hours after electroporation (n = 4 biological replicates, data shown as mean, error bars = SEM). (H) Proliferation in MS5 coculture of *PROM1* KO and control ^CRISPR^*KMT2A*::*AFF1* leukemia cells. Y-axis values expressed as fold change in absolute blast count of *PROM1* KO cells compared to control ^CRISPR^*KMT2A*::*AFF1* cells at each time point (n = 4 biological replicates, error bars = SEM). (I) Top row: survival curves of immunodeficient NSG mice transplanted with *PROM1* KO and control ^CRISPR^*KMT2A*::*AFF1* blasts for 2 CD133^+^ leukemias (n = 3 Cas9 controls and n = 3 *PROM1* KO animals for each leukemia; *P* = .06 and *P* = .02, respectively). Bottom row: CD133 and CD34 status of blast cells recovered from bone marrow of animals at cull.

To assess drug sensitivity profiles, we performed CellTiter-Glo cell-viability assay on FACS CD133^+^ and CD133^–^^CRISPR^*KMT2A*::*AFF1* blasts after treatment with 4 ALL drugs: prednisolone, L-asparaginase, daunorubicin, and vincristine. This showed no significant difference in drug sensitivities between CD133^+^ and CD133^–^ blasts (supplemental Figure 3).

Finally, the functional requirement for CD133 was investigated in a relevant primary human fetal hematopoietic stem and progenitor cell–derived model system.^17^ *PROM1* KO in ^CRISPR^*KMT2A*::*AFF1* ALL led to a significant loss of CD133 expression by flow cytometry (Figure 2F), and an increased proportion of cells in sub-G1 phase 72 hours after KO (Figure 2G; 22.8% vs 7.7%, *P* = .02), suggesting increased apoptosis in *PROM1* KO cells. In keeping with this, *PROM1* KO significantly reduced the proliferative potential of ^CRISPR^*KMT2A*::*AFF1* blasts cultured in vitro (Figure 2H). *PROM1* KO or Cas9-only control ^CRISPR^*KMT2A*::*AFF1* blasts were also transplanted into NSG mice 12 to 18 hours after KO (n = 2 ^CRISPR^*KMT2A*::*AFF1* leukemias, 12 mice). Mice transplanted with *PROM1* KO blasts had a longer survival duration compared to Cas9-only controls (Figure 2I). Leukemias in the Cas9 control mice had a similar proportion of CD133^+^ blasts at cull as the original leukemia, but leukemias derived from *PROM1* KO blasts also demonstrated some CD133 positivity at the point of culling (Figure 2I). The CD133^+^ blasts in *PROM1* KO xenografts probably represent outgrowth of residual blasts which escaped KO (Figure 2F).

Together, these data show that in ^CRISPR^*KMT2A*::*AFF1* ALL, CD133^+^ blasts are enriched for a stem cell-like signature and possess almost all the proliferative potential of the leukemia. Enhancement of leukemic proliferation by CD133 can be seen directly in vitro and indirectly in vivo, with animals receiving CD133^–^ blasts having prolonged survival.

Our studies support a role for CD133 in promoting proliferation and resisting apoptosis in *KMT2A*::*AFF1* ALL blasts. Although immunotherapeutic targeting of functionally neutral markers, such as CD19, has been successful in the treatment of iALL,^20^^,^^21^ treatment evasion by downregulation of the target molecule,^22^ or relapse driven by CD19^–^ upstream progenitors^23^ remains a serious clinical problem. We conclude that in *KMT2Ar* leukemias where CD133 is expressed, there is a strong rationale for targeting this molecule as part of a combination approach.^24^^,^^25^

**Conflict-of-interest disclosure:** T.A.M. and N.C. are paid consultants for and shareholders in Dark Blue Therapeutics Ltd. The remaining authors declare no competing financial interests.

## Supplementary Material

Supplemental Figures, Tables, Methods, and References
