# Supplementary material for: PROM1/CD133 marks a proliferative stem cell-like population of blasts in KMT2A rearranged infant ALL
Source: Blood Adv. 2025 Jul 5;9(18):4607–13. doi: 10.1182/bloodadvances.2024015185 (PMC12455107; doi:10.1182/bloodadvances.2024015185)
Supplement: Supplemental Figures, Tables, Methods, and References [file BLOODA_ADV-2024-015185-mmc1.pdf]

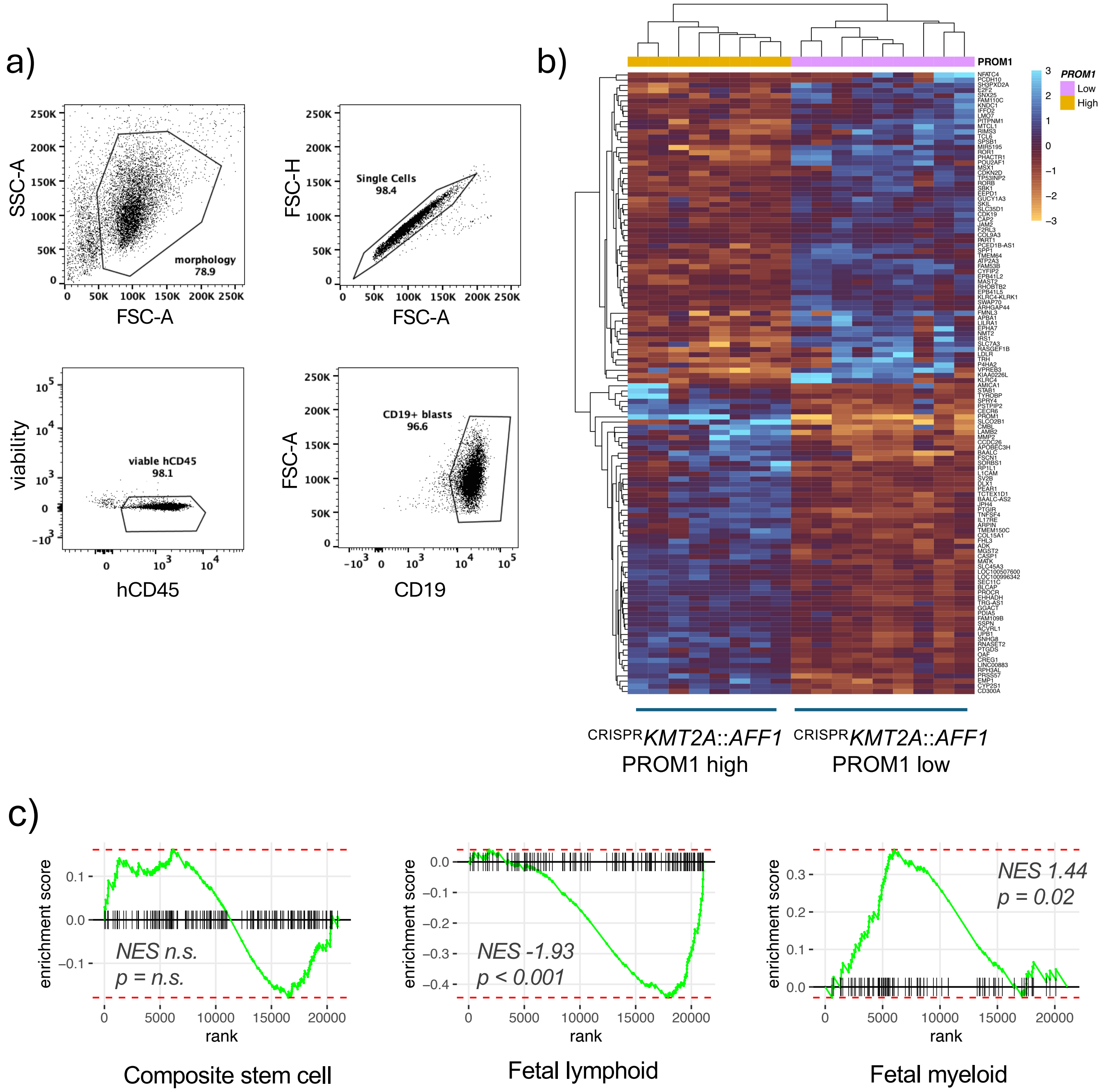

Supplemental Figure 1

a) FACS gating strategy for isolating CD19+ blasts from CRISPR*KMT2A*::*AFF1* leukemias

b) Heatmap of DEGs between *PROM1* high (n = 8) and *PROM1* low (n = 9) CRISPR*KMT2A*::*AFF1* leukemias.

c) GSEA for composite stem cell[19], fetal lymphoid[18] and fetal myeloid[18] geneset, comparing PROM1 low (bottom 20% of PROM1 expression) to PROM1 high (remainder) MLL rearranged infant leukaemias in a published bulk RNAseq dataset[10].

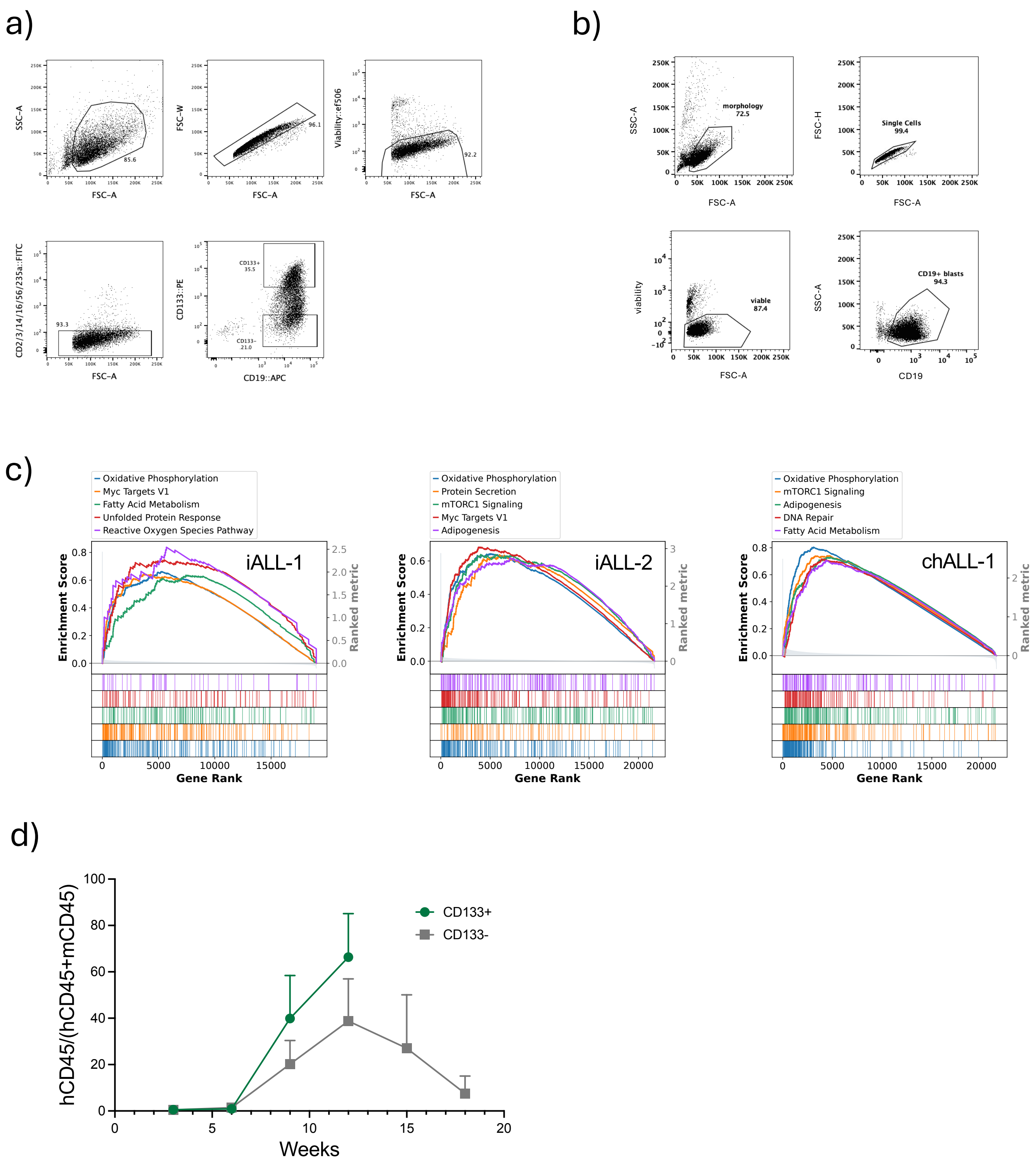

Supplemental Figure 2

a) FACS gating strategy for isolating CD133+ and CD133- blasts from ^{CRISPR}*KMT2A*::*AFF1* leukemias.

b) FACS gating strategy for isolating CD19+ blasts from *KMT2A*::*AFF1* ALL patient samples for single cell transcriptomic assays.

c) Single cell transcriptomic assay: GSEA showing pathways significantly enriched in *PROM1*+ blasts compared to *PROM1*- blasts for each *KMT2A*::*AFF1* ALL patient sample (n=3)

d) Peripheral blood engraftment in NSG mice of CD133+ and CD133- sorted ^{CRISPR}*KMT2A*::*AFF1* blasts (n = 12 CD133+, n=11 CD133-, error bars = SEM)

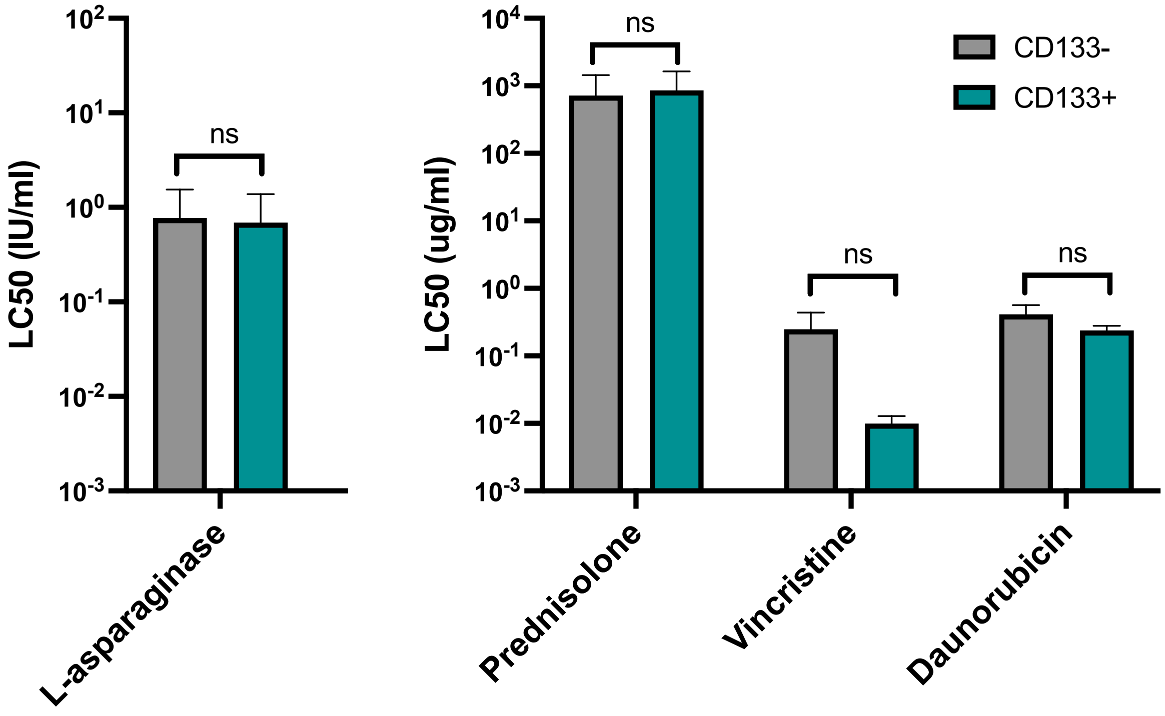

Supplemental Figure 3

Drug sensitivity assays. LC50 values for CD133 positive and negative blasts sorted from [CRISPR]*KMT2A*::*AFF1* leukemias (n = 3 biological replicates, error bars = SEM, ns = not significant by multiple Wilcoxon tests).

Supplemental Tables

sgRNA target	sgRNA sequence
PROM1 +16075896	GCAACAGGGAGCCGAGUACG
PROM1 -16075877	UCGGCUCCCUGUUGCUGCUG
PROM1 -16075896	CUAGCUAUGGCCCUCGUACU

Supplemental Table 1
Single guide RNA (sgRNA) sequences used for CRISPR-Cas9 knockout of *PROM1*

Target	Fluorochrome	Manufacturer	Cat. number	Clone
CD2	PerCP-Cy5.5	Biolegend	300216	RPA-2.10
CD2	FITC	Life Tech	11-0029-42	RPA-2.10
CD3	PerCP-Cy5.5	Biolegend	317336	OKT3
CD3	FITC	Life Tech	11-0037-42	OKT3
CD10	PE-Cy7	Life Tech	25-0106-42	ebioCB-CALLA
CD10	FITC	Life Tech	11-0106-42	ebioCB-CALLA
CD11b	PerCP-Cy5.5	Biolegend	301328	ICRF44
CD14	PerCP-Cy5.5	Biolegend	301824	M5E2
CD14	FITC	Life Tech	11-0149-42	61D3
CD16	PerCP-Cy5.5	Biolegend	302028	3G8
CD16	FITC	Life Tech	11-0168-42	eBioCB16
CD19	APC	Biolegend	302212	HIB19
CD20	eFluor 450	Life Tech	48-0209-42	2H7
CD33	FITC	Biolegend	366620	P67.6
CD34	Brilliant Violet 421	Biolegend	343610	561
CD34	PE-Cy7	Life Tech	25-0349-42	4H11/8G12
CD38	Brilliant Violet 605	Biolegend	303532	HIT2
CD45	Alexa Fluor 700	Life Tech	56-9459-42	2D1
CD45.1 (murine)	APC-Cy7	Biolegend	103116	30-F11
CD56	Brilliant Violet 605	Biolegend	318334	HCD56
CD56	PerCP-Cy5.5	Biolegend	318322	HCD56
CD133	PE	Miltenyi	130-113-108	AC133
CD235a	PerCP-Cy5.5	Biolegend	306614	HIR2
Viability	eFluor fixable 506	eBioscience	65-0866-14	-
Viability	Hoechst 33342	Cayman	15547	-

Supplemental Table 2
Fluorochrome-labelled primary antibodies for flow cytometry and FACS

PROM1* high ^{CRISPR}*KMT2A*::*AFF1					
PROM1	L1CAM	RASL12	EPHB3	MAN1B1-AS1	EMP1
SLCO2B1	ARMCX1	FAM86EP	IRX3	CRADD	NFE2L3
PSTPIP2	RAB13	MAN1C1	IL17RE	IGFBP4	ZNF462
SORBS1	RNASET2	STARD8	LINC00092	MARVELD1	WWC2
SLC45A3	CCDC85B	PLXNA2	LINC00669	ZNF593	RPL38
MGST2	SCRN2	SIRPB2	SLC22A4	IL1RN	GAS2L1
SNHG8	GPRC5C	BAIAP2	MAFB	RNASE2	AGRN
PTGIR	OAF	PHLDB1	DPCD	JPH4	NID1
CEBPB	LOC100507600	PVR	PROCR	AMICA1	C11orf1
MAPK12	SIGLEC12	CCRL2	BNIP3	TNFSF4	RPS29
CD34	LOXL1-AS1	VANGL1	HOMER3	IL15RA	PECAM1
C1QTNF4	FXYD6	KCNK5	CRIP2	TTYH2	MSL3P1
TMEM177	ARL9	MVP	TIFAB	CD14	PTPRG
FHL3	ZNF467	COL6A3	PRSS57	LINC01139	ABR
C9orf89	GPR12	TCTEX1D1	SLC27A2	MYO1B	SIL1
STAB1	EPAS1	TMEM163	MYL5	ABHD14A	EFEMP2
ADAM33	SLCO5A1	TNFAIP8L2	CACNB4	EIF4EBP1	DUSP19
LAMB2	RAB31	CREG1	NPDC1	IFI44	PLVAP
ECHDC2	PLTP	USP18	HILPDA	FAM180B	RHOC
MRPS21	MTMR11	RP1L1	ADK	COL15A1	PLEKHA1
LOXL1	NPM3	MRPS33	ZFP57	CD1E	LRRC75A-AS1
SPRYD4	TMEM173	TMEM150C	TRG-AS1	LYSMD4	ATP8B4
ZBED6CL	ARAP3	CYTIP	SUOX	TMEM144	BAIAP2-AS1
TIE1	CD36	PACSIN1	UBXN10-AS1	TSTD1	LINC00883
CTSW	ARPIN	BLCAP	SEC11C	NPW	LRRC34
LOC100996342	CDYL2	ADAMTS18	PRAM1	MMP2	LOC730102
SHROOM4	CYP2S1	RPL12	PLXDC2	MYO10	IL17RC
MTSS1L	NUDT14	MT1X	CASP1	EPHA2	F13A1
TYROBP	PVRL2	ZDHHC1	CDKN2A	CX3CR1	IGFBP3
AIF1L	ITGAL	MAP3K13	SLC44A5	LMNA	SMIM6
CYFIP1	KCNE5	PMP22	SVIL-AS1	CD300A	
CECR6	DLX1	LGALS3	ASGR1	ARHGAP22	
TNFAIP2	RPL36	FAM109B	ACVR2B-AS1	GNPDA1	
SERPING1	IL3RA	CTDSPL	RASGRP3	LRRC61	
CDH5	LMO2	CCDC26	LYZ	MMP15	
H2AFY	RBFOX2	TPM2	SV2B	PKP1	
GPRC5B	PTGDS	DHX32	C20orf27	GGACT	
NDFIP1	ITGA3	PCDH8	RFX8	SPRY4	

PROM1* low ^{CRISPR}*KMT2A*::*AFF1		
BEND4	FAM177B	C10orf12
EPB41L2	SBK1	TEAD1
SWAP70	ZNF318	TBX20
VAT1L	EIF5A2	
APBA1	RASGRP1	
SESTD1	TDRKH	
FAM53B	RIMS3	
EEPD1	TLR9	
ATP2A3	KCTD7	
EPB41L5	SH3PXD2A	
DUSP1	OTUD1	
KIAA0226L	ABCA12	
TMEM64	MSX1	
SPP1	PTPRC	
PHACTR1	B3GNT5	
ZNF595	RASGEF1B	
KLRC4	FOS	
RORB	KLRC3	
ROR1	PLEKHH2	
SNX25	LMO7	
FCHO1	GUCY1A3	
FAM110C	EPHA7	
POU2AF1	CAP2	
SLC35D1	AXIN2	
BVES	CTD-2201I18.1	
CELSR2	CYFIP2	
HEXIM1	PRKG2	
RHOBTB2	NMT2	
LOC613266	KIF13A	
PIM1	JAM2	
KLRC4-KLRK1	MAP7D2	
EGLN1	CPEB3	
KNDC1	IRS1	
SLC7A3	KIAA2018	
ARHGAP44	MME	
TBC1D32	BEX4	
LILRA1	PRPF40B	
MIR5195	NRBP2	

Supplemental Table 3
DEGs between *PROM1* high and *PROM1* low ^{CRISPR}*KMT2A*::*AFF1* ALL, fold change >2, FDR <0.05 (n=8 *PROM1* high, n=9 *PROM1* low).

**CD133+ ^{CRISPR}*KMT2A*::*AFF1* blasts**				
PROM1	NEURL1B	FOXM1	TMPO-AS1	TMEM150C
TOP2A	COL5A1	FXYD6	FLT4	LAMB2
MKI67	CENPF	ERG	HMMR	TJP1
RACGAP1	TIMP2	MELK	MYO5C	RASIP1
NUSAP1	DLGAP5	UBE2T	DYRK3	ARAP3
HJURP	TPX2	GGH	TSPAN15	VASH1
KIF11	SHCBP1	DLC1	CASKIN2	LGR6
UBE2C	AGRN	ASPM	JPH1	LRP11
KIFC1	LIMS1	CDH5	HMGA2	CENPI
ARHGAP11A	WWTR1	KIF14	BGN	SLC9A3R2
CKAP2L	CKAP2	SPAG5	MUC4	PDE4B
CDCA8	TTK	TACC3	ESCO2	SH3RF3
CCNF	PCDH12	FANCD2	EFNA1	KIF24
ANLN	NCAPG	EPAS1	CD59	MAGI3
NDC80	ECT2	FBXO5	PALM	C21orf58
CCNA2	E2F8	SMTN	GNG11	
INCENP	ELFN2	FAM83D	SGOL2	
PRC1	CDCA5	GAS2L3	CEP55	
CIT	MDK	SOX7	SERPINB9	
PLK1	CENPE	N4BP3	ECM1	
CDK1	KIF18B	TRIM59	FAM129A	
FAM64A	AIF1L	AURKA	STK32B	
NEK2	GTSE1	KIF4A	OSBPL10	
CDCA2	CORO2B	GPSM2	SPC24	
RRM2	RAD51AP1	KIF15	CDKN3	
SLCO2B1	AKAP12	IQGAP3	RAB15	
KIF20A	DYSF	DOCK6	POU4F1	
CDKN2C	SPC25	GSG2	FBXO43	
TICRR	STIL	PSRC1	PALD1	
DEPDC1	PXN	BRIP1	TCF7	
NCAPH	CENPL	MYO10	LOXL1	
KIF2C	TK1	NEIL3	ZNF467	
PLK4	KIF18A	MYO7A	LOC100288637	
SLC16A2	PEAR1	BUB1B	CDC42EP5	
ESPL1	CDCA3	OAS3	CDC42EP4	
KIF23	AURKB	CACNA2D2	CDC25C	
CASC5	ANTXR1	NRIP1	ERCC6L	
BCL6B	CENPA	UACA	CLEC14A	

**CD133- ^{CRISPR}*KMT2A*::*AFF1* blasts**	
CD52	RGS1
ITGB7	CTTN
CD24	GLDC
RHOB	DENND1C
CLEC2D	LPXN
CD72	C16orf74
NCF1	MXD4
SCIMP	PARP15
MYL4	HLA-DMB
TENM4	SELL
GPR183	IGLL5
FGD2	FKBP4
SCN3A	NR4A2
CHST2	SPIB
AK5	VPREB3
ADAM23	HCK
PDGFRB	HCST
HVCN1	NMB
TCL1B	HLA-DQA2
SAMHD1	MAFB
NT5E	TLR7
FAM129C	PTGER4
ANXA1	SNX25
KLF2	RASGRP1
ADAM19	ADARB1
HLA-DQA1	TNFSF9
GBP2	CST7
VCAN	P2RX5
SEL1L3	SMIM14
MPEG1	KCNJ16
FRY	RASAL1
IKZF3	IFNLR1
ARL4C	LOC101927412
BMF	IGFLR1
LBH	GPR18
NCF1C	LIMK1
SGPL1	MYO7B
CDHR3	

Supplemental Table 4
DEGs between FACS-sorted CD133+ and CD133- blasts from the same ^{CRISPR}*KMT2A*::*AFF1* ALL (n=3)

**sample**	**sc RNA-seq**		**protein expression**
	PROM1+ (cell count)	*PROM1*- (cell count)	% of blasts CD133+
iALL-1	2227	1074	67.1
iALL-2	3612	509	73.2
chALL-1	3211	1762	75.9
chALL-2	13	2519	0.1

Supplemental Table 5
Single cell gene expression data and flow cytometry data showing no. cells expressing *PROM1* gene or not in each patient sample; and % of CD19+ blasts that were CD133+ by flow for the same samples

Supplemental Methods

Patient samples and consent

Donated fetal tissue was provided for the purposes of this research by the Human Developmental Biology Resource (HDBR, www.hdbr.org), regulated by the UK Human Tissue Authority (HTA, www.hta.gov.uk) and covered under ethics (REC: 18/NE/0290 and 18/LO/0822). Informed consent was obtained from all participants, who donated human fetal tissue for research. ALL patient samples were obtained from VIVO Biobank, UK under their ethics approval (REC: 23/EM/0130), and from Our Lady's Children's Hospital, Crumlin, Ireland (under our ethics approval: REC: 21/LO/0195). Informed consent was obtained from all participants or those with parental responsibility. Infant and pediatric KMT2A-r ALL patients being treated at Great Ormond Street Hospital for Children, London had immunophenotypic analysis performed as part of their diagnostic workup and the data shared after informed consent was obtained from all participants or those with parental responsibility. All patient samples/data were anonymized at source, assigned a unique study number and linked.

Animals

Experiments were performed under the Animal (Scientific Procedures) Act 1986 with institutional approval by local ethical review body. Experimental animals were 8–12-week-old NOD.Cg-Prkdc^{scid} Il2rg^{tm1Wjl}/SzJ (NSG) mice.

^{CRISPR}KMT2A::AFF1 leukemia blasts

KMT2A::AFF1 leukemias were generated from primary human fetal liver HSPC, as previously described.[2] ^{CRISPR}KMT2A::AFF1 leukemia was generated from HSPC from 13-20 pcw primary human FL or fetal bone marrow as described in[2]. CRISPR edited fetal HSPC were used for in vivo serial transplantation experiments. BM harvested from primary, secondary and tertiary NSG mice with ^{CRISPR}KMT2A::AFF1 leukemia were cryopreserved in liquid nitrogen vapor phase. Experiments were performed using CD19+ blasts from these BM cells.

Flow cytometry and cell sorting

Cells were stained with fluorochrome-conjugated monoclonal antibodies for 20 minutes at 4°C and analyzed using a BD LSR Fortessa X-50/X-20 flow cytometer. For cell sorting a BD FACSAria Fusion machine was used with a 100 µm nozzle and four-way purity setting. Antibodies are listed in Supplementary Table 2. Data acquisition was done with BD FACSDiva (v8.0.2) and subsequent analysis with FlowJo (v10.10.0). Positive and negative

gates were established using fluorescence-minus-one controls, unstained cells or isotype controls as appropriate. CountBright Absolute Counting Beads (Thermo Fisher) were used for quantitation.

Cell cycle analysis

Stained cells were fixed and permeabilised with Intrastain (Dako) following the manufacturer's protocol. Subsequent staining was performed with Alexa Fluor 700 anti-human Ki-67 antibody (BioLegend) and then Hoechst 33342 (1.25µg/mL, Cayman 15547) for 20 minutes at 4°C before analysis by flow cytometry as previously described.[3]

CellTiter Glo assays for drug response profiling

Cryopreserved blast cells harvested from the spleen of ^{CRISPR}*MLL-AF4*+ mice were FAC-sorted to isolate CD133+ and CD133- blasts. These were assayed *in vitro* for their response to prednisolone, vincristine, daunorubicin and L-asparaginase using the CellTiter-Glo 2.0 assay (Promega, G9241). Briefly, cells were resuspended at 100,000 cells/ml for CD133+ blasts or 400,000 cells/ml for CD133- blasts in RPMI supplemented with 15% heat-inactivated FBS, 100U/ml Penicillin, 100µg/ml Streptomycin. In opaque flat-bottom 96-well plates, 100µl cell suspension ($0.1x10^5$ cells for CD133+ blasts or $0.4x10^5$ cells for CD133- blasts) was treated with a range of concentrations of prednisolone (Sigma, final concentrations: 0.05-900µg/ml), L-asparaginase (Cambridge Biosciences, final concentrations: 0.0003-10IU/ml), vincristine (Cambridge Biosciences, final concentrations: 0.05-50µg/ml) or daunorubicin (Cambridge Bioscience, final concentrations: 0.002-2µg/ml) based on previously published studies. For prednisolone, DMSO was added to untreated control wells while water was added to untreated control wells for all other drugs.
After 48hr incubation at 37°C and 5% CO_2, CellTitre-Glo 2.0 reagent was added to each well. The luminescence of each well was measured on a SPECTROstar Nano (BMG Labtech) microplate reader. For each drug concentration, leukemia cell survival (LCS) was calculated by the following equation: LCS = (luminescence treated well / luminescence untreated well) x 100%. Drug resistance was expressed by the LC50, the drug concentration lethal to 50% of the cells.

Bulk RNA-sequencing

RNAseq libraries were prepared from $1\text{-}3x10^5$ FACS-sorted leukemic cells. RNA extraction was performed using a RNeasy Micro Kit (Qiagen). Library preparation and sequencing were performed as previously described.[2]

Single-cell RNA-sequencing

Single cell multiomic profiling was carried out for four *KMT2A-AFF1+* ALL patient samples. Cryopreserved BM cells from the patients were thawed, and 16,000 live CD19+ blasts from each patient sample were FACS-sorted for single nucleus multiome analysis (Suppl. Fig 2b). Cells from 2 patient samples were pooled, and nuclei processed according to manufacturer's instructions (Chromium 10x: CG000338). Nuclei from 2 pooled samples were loaded on one lane of the Chromium 10x platform. Sequencing was performed by on a NovaSeq 6000, gene expression libraries were sequenced on a PE150 S4 flow cell and the ATAC-seq modality on a SP PE50 flow cell.

Bioinformatic analysis

Bulk RNA-seq data quality was assessed with fastqc (v0.11.9), quality filtered and trimmed using TrimGalore (v0.6.10), aligned to the hg38 reference genome with STAR (v2.7.11a) and transcript reads counted with featureCounts (v2.0.6). Differential gene expression analysis was performed in R (v4.2.2)/Rstudio (v2023.12.1) with DESeq2 (v1.40.1) or limma (v3.2.1). Geneset enrichment analysis was performed using the fast GSEA implementation in fgsea (v1.26.0) with a false discovery rate of 0.05. Gene sets were obtained from MSigDB or manually constructed from published work where indicated.

Single Cell multiome data was aligned using Cell Ranger ARC (v2.0) and ambient RNA was removed using CellBender (v0.3.2). Samples were deconvoluted using souporcell (v2.5) and sample identity was assigned by determination of biological sex using the ratio of XIST expression compared to counts for all genes on chrY. Gene expression data was processed using scanpy (v1.10.2). Low quality cells were removed, counts were normalised, and dimensionality reduction (UMAP) was performed following the best practices workflow.[4] *PROM1* positive cells were defined as those with log1p(normalised PROM1 expression) > 0.15. GSEA analysis was performed using GSEApy (v1.1.3) using the MSigDB Hallmark 2020 database.

Additional statistics were calculated using Graphpad Prism (v10.1.1) and the survival R package (v3.6.4).

Data availability

All RNAseq data has been deposited at GEO accession IDs GSE278786 and GSE295316.

REFERENCES

1. Greil J, Gramatzki M, Burger R, et al. The acute lymphoblastic leukaemia cell line SEM with t(4;11) chromosomal rearrangement is biphenotypic and responsive to interleukin-7. *Br J Haematol*. Feb 1994;86(2):275-83. doi:10.1111/j.1365-2141.1994.tb04726.x
2. Rice S, Jackson T, Crump NT, et al. A human fetal liver-derived infant MLL-AF4 acute lymphoblastic leukemia model reveals a distinct fetal gene expression program. *Nat Commun*. Nov 25 2021;12(1):6905. doi:10.1038/s41467-021-27270-z
3. O'Byrne S, Elliott N, Rice S, et al. Discovery of a CD10-negative B-progenitor in human fetal life identifies unique ontogeny-related developmental programs. *Blood*. Sep 26 2019;134(13):1059-1071. doi:10.1182/blood.2019001289
4. Heumos L, Schaar AC, Lance C, et al. Best practices for single-cell analysis across modalities. *Nat Rev Genet*. Aug 2023;24(8):550-572. doi:10.1038/s41576-023-00586-w
